# Factors associated with post-traumatic stress disorder in family caregivers of psychotic patients

**DOI:** 10.1192/j.eurpsy.2021.1204

**Published:** 2021-08-13

**Authors:** M. Daoud, N. Charfi, S. Omri, R. Feki, N. Smaoui, M. Maalej Bouali, L. Zouari, J. Ben Thabet, M. Maalej

**Affiliations:** 1 Department Of Psychiatry “c”, Universty Hospital of Hedi Chaker, sfax, Tunisia; 2 Psychiatry C Department, Hedi chaker University hospital, sfax, Tunisia

**Keywords:** Aggression, caregivers, post-traumatic stress disorder, psychosis

## Abstract

**Introduction:**

An association can be found between patient with psychosis and perpetrating acts of violence. So, the caregiving role can impact negatively on psychosis carer psychological health and wellbeing.

**Objectives:**

The aim of this study was to identify the factors associated with post-traumatic stress disorder (PTSD) in family caregivers of psychotic patients following exposure to aggression.

**Methods:**

This cross-sectional study was carried out involving 95 family caregivers of psychotic patients followed in psychiatry. Data were gathered from caregivers about their experiences in providing care. Sociodemographic and clinical data of patients were collected from medical records.We used the perceptions of prevalence of aggression scale (POPAS) to measure the frequency and severity of aggression directed at the respondent in the past and the Impact of Event Scale-Revised (IES-R) to evaluate PTSD.

**Results:**

The caregivers were male in 51.6% and with low educational level in 46.3% of cases. A rate of 75.8% of caregivers reported experiencing moderate to severe levels of aggression. More than a half of caregivers (54.7%) reported potentially significant levels of PTSD. Decreased contact with patient (p=0.01), male gender (p=0.00), older age (p=0.00), living far from patient (p=0.00), parent relationship of caregivers (p=0.00), diagnosis of schizophrenia or schizoaffective disorder (p=0.00) and poor adherence to treatment (p=0.00) in affected relatives were associated with the presence of PTSD following exposure to moderate to severe aggression.

**Conclusions:**

These findings highlight the need for interventions to promote family psychoeducation and to provide psychosocial support for caregivers of patients in order to prevent the traumatic impact of violence on them.

